# Recent Advances in Self-Powered Wearable Sensors Based on Piezoelectric and Triboelectric Nanogenerators

**DOI:** 10.3390/bios13010037

**Published:** 2022-12-27

**Authors:** Arash Rayegani, Mohammadmohsen Saberian, Zahra Delshad, Junwei Liang, Muhammad Sadiq, Ali Matin Nazar, Syed Agha Hassnain Mohsan, Muhammad Asghar Khan

**Affiliations:** 1 Department of Civil Engineering, Sharif University of Technology, Tehran 1458889694, Iran; 2 Department of Computer Science and Engineering, Qatar University, Doha P.O. Box 2713, Qatar; 3 Department of Nursing, Kashan Branch, Islamic Azad University, Kashan 8715998151, Iran; 4 College of Software Engineering, Shenzhen Institute of Information Technology, Shenzhen 518172, China; 5 Shenzhen Institute of Information Technology, Shenzhen 518172, China; 6 The Zhejiang University-University of Illinois at Urbana-Champaign Institute, Zhejiang University, Haining 314400, China; 7 Optical Communications Laboratory, Ocean College, Zhejiang University, Zheda Road 1, Zhoushan 316021, China; 8 Hamdard Institute of Engineering and Technology, Hamdard University, Islamabad 700081, Pakistan

**Keywords:** triboelectric nanogenerators (TENG), piezoelectric nanogenerators (PENG), self-powered wearable sensors

## Abstract

Early clinical diagnosis and treatment of disease rely heavily on measuring the many various types of medical information that are scattered throughout the body. Continuous and accurate monitoring of the human body is required in order to identify abnormal medical signals and to locate the factors that contribute to their occurrence in a timely manner. In order to fulfill this requirement, a variety of battery-free and self-powered methods of information collecting have been developed. For the purpose of a health monitoring system, this paper presents smart wearable sensors that are based on triboelectric nanogenerators (TENG) and piezoelectric nanogenerators (PENG), as well as hybrid nanogenerators that combine piezoelectric and triboelectric nanogenerators (PTNG). Following the presentation of the PENG and TENG principles, a summary and discussion of the most current developments in self-powered medical information sensors with a variety of purposes, structural designs, and electric performances follows. Wearable sensors that generate their own electricity are crucial not only for the proper development of children and patients with unique conditions, but for the purpose of maintaining checks on the wellbeing of the elderly and those who have recently recovered from illness, and for administering any necessary medical care. This work sought to do two things at once: provide perspectives for health monitoring, and open up new avenues for the analysis of long-distance biological movement status.

## 1. Introduction

**Figure 1:**
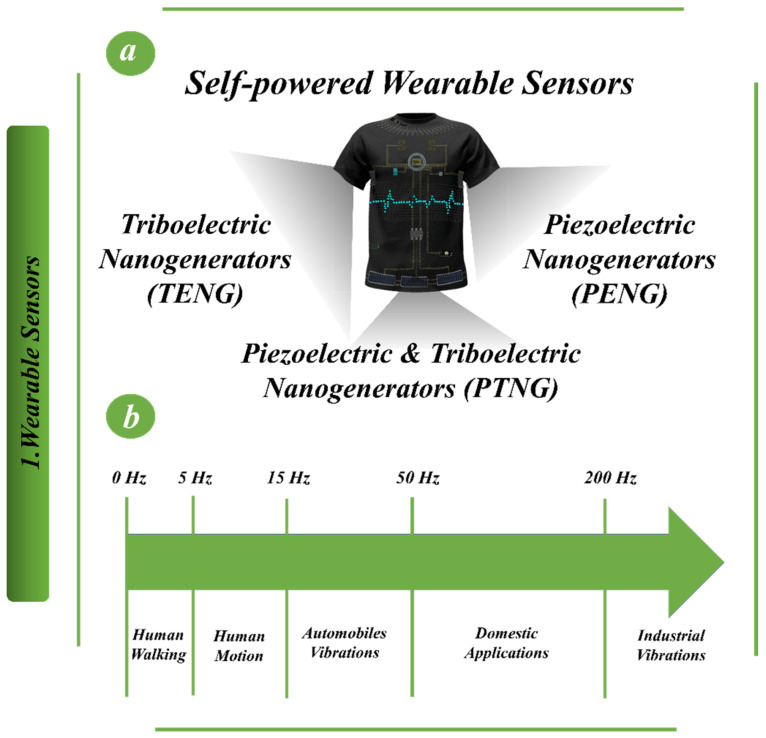
(**a**) Self-powered wearable sensors based on piezoelectric and triboelectric nanogenerators (**b**) Frequency levels of common mechanical energy sources.

Due to their qualities of high sensitivity [1] high resolution, and low cost [2], multifunctional flexible sensors have recently been widely used in human health detection, intelligent robotics and other disciplines. In recent years, the gathering and analysis of health data have been greatly aided by wearable technology [3,4,5,6]. It is a useful technique for the early identification, diagnosis, and treatment of diseases [7,8,9,10]. As an illustration, long-term monitoring of bodily indicators such as EKG, heartbeat, blood pressure and pulse is considered in the analysis and diagnosis of cardiovascular illnesses [11]. However, conventional electrochemical batteries are the major source of the electricity needed for wearable technology [12,13,14,15,16,17,18]. They not only add size and discomfort to worn sensors, but they also necessitate frequent recharging or regular replacement. Various types of these sensors may be employed in a variety of applications, including engineering [19,20]. Numerous wearable energy harvesters have been developed using diverse concepts, including electrostatic [21,22], electromagnetic, thermoelectric, piezoelectric [12,23] and triboelectric [24,25,26] power. Self-powered energy harvesters may transform many forms of ambient energy into electrical energy [21,27,28,29,30,31,32,33,34,35] and, to a certain extent, can replace batteries. In the most recent research, ultrasound in vivo can be captured using capacitive triboelectric electrets which can also power medical implants [30,36,37,38,39,40,41,42,43]. The advantages of self-powered energy harvesters over batteries are low cost, compact size, light weight and environmental friendliness, which prevent missing important abnormal medical signals when the battery is depleted [44,45,46,47,48,49,50,51,52,53]. The energy that is harvested can come from the movements of body as well as the surrounding environment, such as motion energy from routine motions [54], human physiological processes [23], the energy of the sun and artificial light and the temperature of the human body [55,56,57,58]. Drawing on a number of techniques, including the triboelectric effect [59,60], piezoelectric effect, Seebeck effect, and photovoltaic effect, these energies can be transformed into electrical energy. Figure 1a shows the purpose of this review is to summarize various types of self-powered medical information sensors based on the triboelectric nanogenerators (TENG), piezoelectric nanogenerators (PENG) and hybrid piezoelectric and triboelectric nanogenerators (PTNG) [27,39,61,62,63,64,65,66,67,68]. Figure 1b illustrates the approximate working-frequency levels of common mechanical energy sources that the frequency levels of human activities are between 0 to 15 Hz, which is important for wearable self-powered sensors [65,66,67,68]. With the rapid development of advanced materials and nanotechnology, sensor technology has advanced to a very high level. However, the majority of them still rely on external power sources, such as batteries, which can lead to problems as they are difficult to track, recycle, and miniaturize. They also pose potential environmental pollution and health risks. Although the researcher may use a wireless recharging system for the replacement of a battery, this may bring serious problems if the technician misses the track during the recharging time. Traditional sensors must be activated by an external power source, and because they are widely dispersed and operate intermittently, they consume a lot of energy, which makes it difficult to create applications that are sustainable, healthy and green. However, self-powered sensors based on triboelectric nanogenerators (TENG) and piezoelectric nanogenerators (PENG) can generate electrical energy for storage by themselves by harvesting energy from their surroundings. Additionally, sensors can be self-powered, which is essential for smart homes, smart wearable technology, and bio-medicine.

This article provides an overview of self-powered wearable sensors based on piezoelectric and triboelectric nanogenerators for healthcare systems. Section 2 describes the principle of piezoelectric and triboelectric nanogenerators. Section 3 provides a brief overview of self-powered wearable sensors based on triboelectric nanogenerators. Section 4 discusses wearable sensors based on piezoelectric nanogenerators. Section 5 describes the self-powered sensors based on hybrid piezoelectric and triboelectric nanogenerators. Section 6 provides a brief review of challenges, perspectives and insight for self-powered wearable sensors.

## 2. Principle of Piezoelectric and Triboelectric Nanogenerators

**Figure 2:**
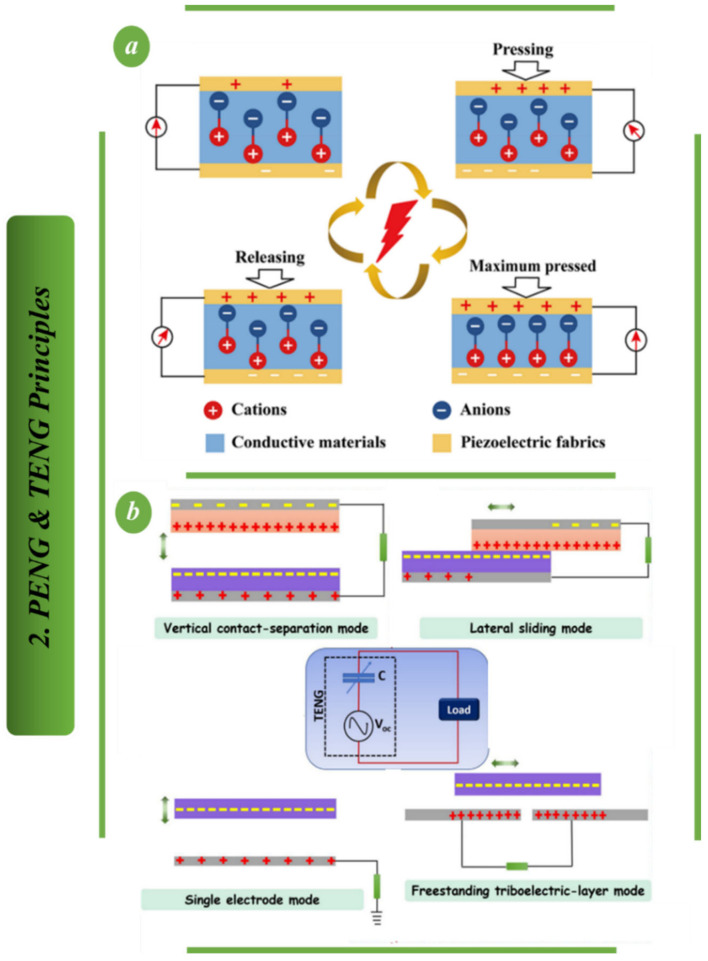
Principle of (**a**) piezoelectric [81] and (**b**) triboelectric nanogenerators [82].

Figure 2 depicts the fundamental form of both piezoelectric and triboelectric nanogenerators. PENGs typically incorporate electrodes and piezoelectric materials [69]. After polarization, when the PENGs are deformed, a charge with the opposite sign emerges on both the top and bottom sides of the piezoelectric materials that correspond to those surfaces [70,71]. Figure 2a provides a diagrammatic depiction of the process by which the PENGs generate electricity when their buttons are pressed and then released [72,73,74,75,76]. Even if cations and anions are both under stress, the equivalent canters of charge for both types of ions remain at the same location. Polarization, as well as the creation of an electric current, are neither present nor occurring [77]. When external forces are applied to the nanogenerator, it results in a contraction of the volume and a stretching in the opposite direction [78]. As a direct consequence of this, the canters of charge of the cations and anions move, electric dipoles are generated, a piezoelectric potential is developed between the two electrodes and a new balancing state is produced [79]. There are four distinct modes of operation for the triboelectric nanogenerators that are depicted in Figure 2b. These include the vertical contact-separation mode, the lateral sliding mode, the single electrode mode and the freestanding triboelectric layer mode [80].

## 3. Self-Powered Wearable Sensors Based on Triboelectric Nanogenerators

**Figure 3:**
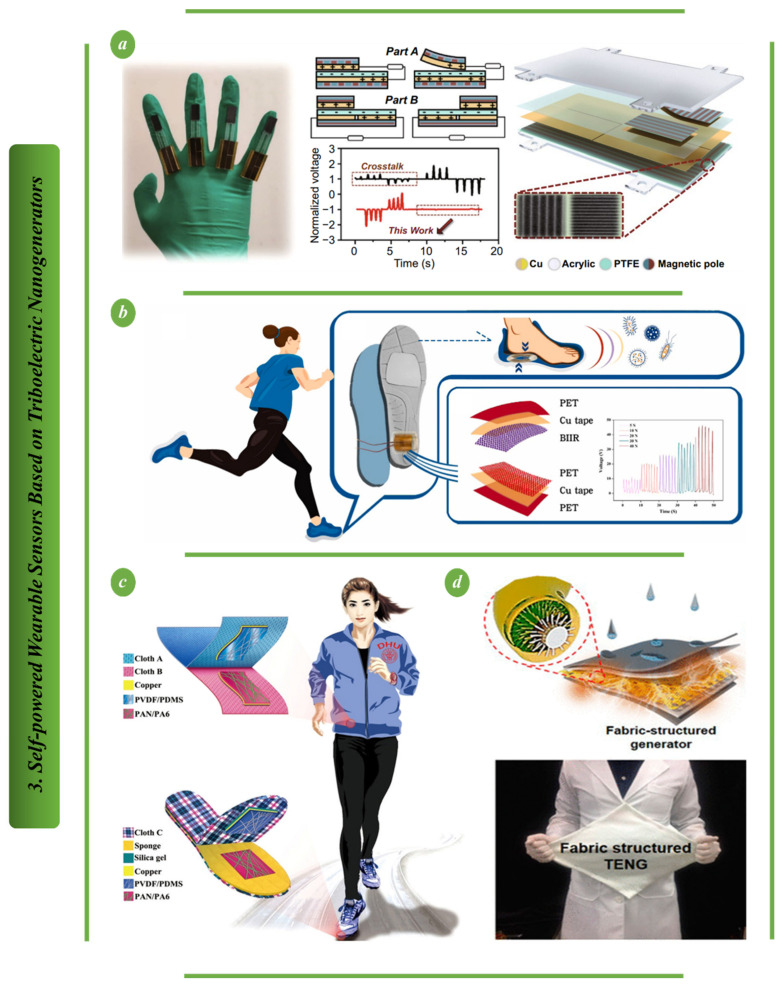
Self-powered wearable sensors based on TENG: (**a**) Multilayer structure of the Ma-s-TS [83]. (**b**) A schematic representation of a triboelectric nanogenerator that was built using bromobutyl rubber (BIIR) and PET [84]. (**c**) Fabricating an NM-TENG for use in wearable electronics [85]. (**d**) Depicting the stages of the FTENG’s manufacturing process [86].

**Figure 4:**
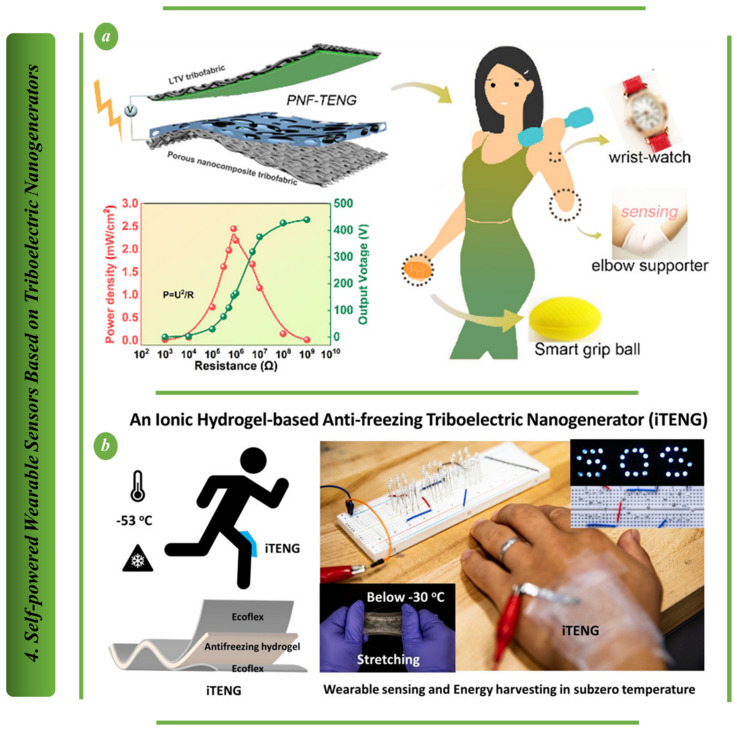
Self-powered wearable sensors based on TENG: (**a**) The PNF-TENG is a self-powered sensor that can also function as a wearable power source [87]. (**b**) The interfacial bonding structure of antifreeze-stretchable iTENG [88].

Self-powered wearable sensors based on triboelectric nanogenerators (TENG) are described in detail in Figure 3 and Figure 4. A sliding triboelectric sensor with magnetic array assistance is shown in Figure 3a. When human hands and robotic hands interact in real-time through gestures, the sensor responds with positive or negative pulse signals depending on whether the finger is flexed or extended.

By monitoring the number of pulses in a given amount of time, one can get a real-time read on the magnitude, velocity and orientation of a user’s finger motions. The measurable pulses are produced in large part by the magnetic array. The system’s longevity, low-speed signal amplitude and stability are all improved by the two components of the magnetic array that were specifically built to convert sliding motion into contact separation and restrict the sliding pathway, respectively [83]. Figure 3b shows triboelectric nanogenerators made from flexible bromobutyl rubber (BIIR) and PET using a variety of film creation and surface roughening processes to create arched vertical contact-separation structures. In order to get the best power generating results, the treatment and assembly methods of the friction materials were investigated [84]. By creating a wearable triboelectric nanogenerator (NM-TENG) made of a nanofibrous membrane, Figure 3c shows that next-generation wearables will require a power source that can convert human biomechanical energy into electricity while being lightweight, adaptable and sustainable. The electro spun nanofibrous membranes’ polarity, mechanical strength and surface hydrophobicity were improved, leading to improved device output performance, robustness and operability even in high environmental humidity [85]. Highly-flexible 2D fabrics are shown in Figure 3d acting as a wearable triboelectric nanogenerator to power wearable electronic devices. In the beginning, fibers were made, primarily from Al wires and PDMS tubes with a high-aspect-ratio nanotextured surface and vertically aligned nanowires. With this design, the nanogenerator produced a steady output performance even when it was severely strained. The FTENG was also used in applications for elbow-mounted power apparel and footstep-driven large-scale power mats for walking. This method is thought to offer a good contender for a high-performance and reliable nanogenerator that will eventually power wearable devices on its own [86]. 

Figure 4a depicts how to create a porous nanocomposite fabric (PNF) with a high capacity for charge accumulation using a simple dry-casting technique, and how to use it as a tribopositive material to create stylish wearable triboelectric nanogenerators (abbreviated as TENGs). This gadget is an effective and dependable green wearable power source as it can power commercial wristwatches, light LED arrays, and charge a variety of capacitors. Additionally, a grip ball and an elbow supporter based on PNF-TENG are presented as self-powered sensors to provide real-time detection of human activities during sports exercises. This research suggests a green wearable triboelectric nanogenerator fabric as an efficient tribopositive material, confirms the viability of creating green wearable power sources and sensors and offers fresh insights into their design [87]. A freeze-tolerant ionic hydrogel and a dielectric elastomer are used as the electrode and electrification layer, respectively, in the low-cost, highly stretchy and antifreezing ionic triboelectric nanogenerator (iTENG) shown in Figure 4b. The iTENG design delivers a special blend of benefits, including strong hydrogel-elastomer bonding, great stretchability (300%), and excellent tolerance to extremely low temperatures. The iTENG in this setup was able to withstand cyclic compression (2000 cycles) without experiencing any electrical degradation [88]. This occurred because of the hydrogel and elastomer’s impressive interfacial adhesion.

**Figure 5:**
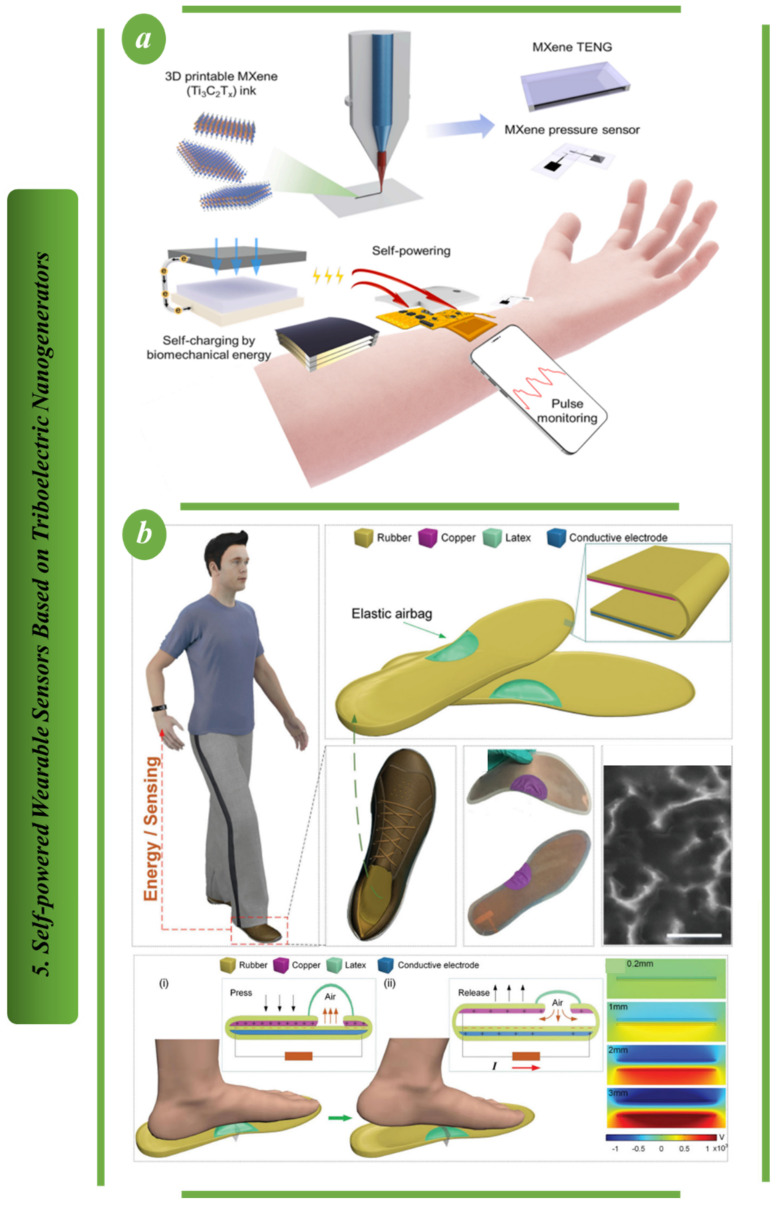
Self−powered wearable sensors based on TENG: (**a**) the design principal of MXene−based TENG (M−TENG) [89]. (**b**) Structural design of the TENG-based system for gathering biomedical energy [90].

Figure 5a depicts the proposal, development, characterization and validation of a novel, self-powered, MXene-based, 3D-printed, flexible and integrated wearable system for continuous, real-time physiological biosignal monitoring. This structure is a fully self-powered RAP monitoring structure that demonstrates wireless data and power transmission via near-field communication. This is the first report of a wearable system for continuous and real-time physiological biosignal monitoring powered entirely by human motion, indicating a promising future in the field [89]. Figure 5b depicts a waterproof and high-performance triboelectric nanogenerator (TENG)-based insole designed to capture human motion energy for the long-term operation of portable devices. To collect mechanical energy, an airtight-cavity-airbag structural insole based on a TENG was designed in the protocol. The contact and separation of triboelectric layers were driven by an elastic airbag through their corresponding expansion and contraction. The findings point to a wide range of potential applications, from powering personal sensing devices to the Internet of Things [90].

**Figure 6:**
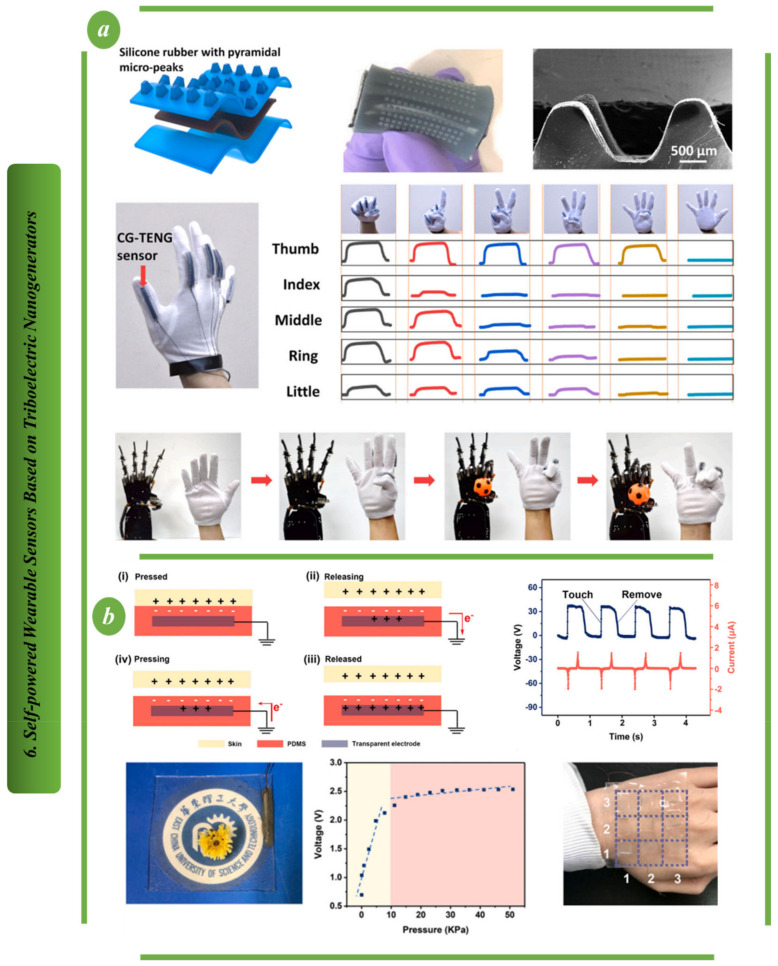
Self−powered wearable sensors based on TENG: (**a**) The design principal of CG−TENG sensor [91]. (**b**) Structural design and output voltage of MAMP−TENG [92].

Figure 6a shows a TENG made from coffee grounds that has the advantages of being inexpensive and environmentally friendly, and having excellent stretchability, deformability and durability. It is also useful for mechanical energy harvesting and self-sufficient pressure sensing, which includes motion monitoring, emulation and acting as a smart human–machine interface. These findings offer a promising framework for the creation of eco-friendly wearable technology and intelligent user interface systems [91]. Figure 6b illustrates a stretchable and transparent TENG based on a Mxene-AgNWs-Mxene-polyurethane nanofibers (MAMP) electrode that exhibits excellent optoelectronic properties with low sheet resistance. Because it combines a motion sensor and a sustainable power source, its potential for use in wearable technology and human–machine systems is high [92].

## 4. Self-Powered Wearable Sensors Based on Piezoelectric Nanogenerators 

**Figure 7:**
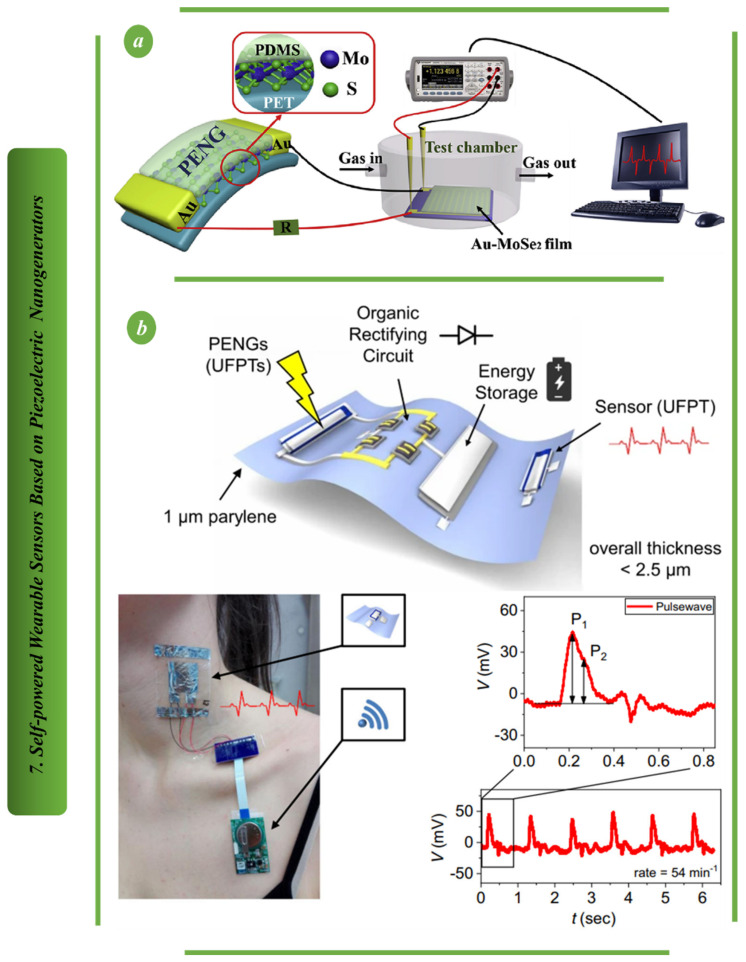
Self−powered wearable sensors based on PENG: (**a**) The design principal of self−power sensor based on flexible piezoelectric nanogenerator [93]. (**b**) Structure of biomedical sensor based on ultraflexible piezoelectric energy harvesting and sensing device [94].

In Figure 7a, a novel flexible piezoelectric nanogenerator (PENG) based on two-dimensional (2D) semiconductor MoS2 flake was used to power a room temperature Au-MoSe2 composite ammonia (NH3) sensor for the first time. Additionally, this design illustrated a MoS2-based PENG device attached to a person’s body for the purpose of harvesting a variety of body motion energy, showcasing its strong potential for use in wearable technology [93]. For imperceptible sensing and energy harvesting systems, ferroelectric polymer transducers and organic diodes are shown in Figure 7b. They are integrated on ultrathin (1-m) substrates, giving them excellent flexibility. Simulations demonstrate that the use of an ultrathin substrate, which enables mounting on 3D-shaped objects and stacking in multiple layers, significantly improves the sensitivity of ultraflexible ferroelectric polymer transducers. In fact, ultraflexible ferroelectric polymer transducers form invisible wireless e-health patches for accurate pulse and blood pressure monitoring due to their improved sensitivity to strain and pressure, quick response and excellent mechanical stability [94].

**Figure 8:**
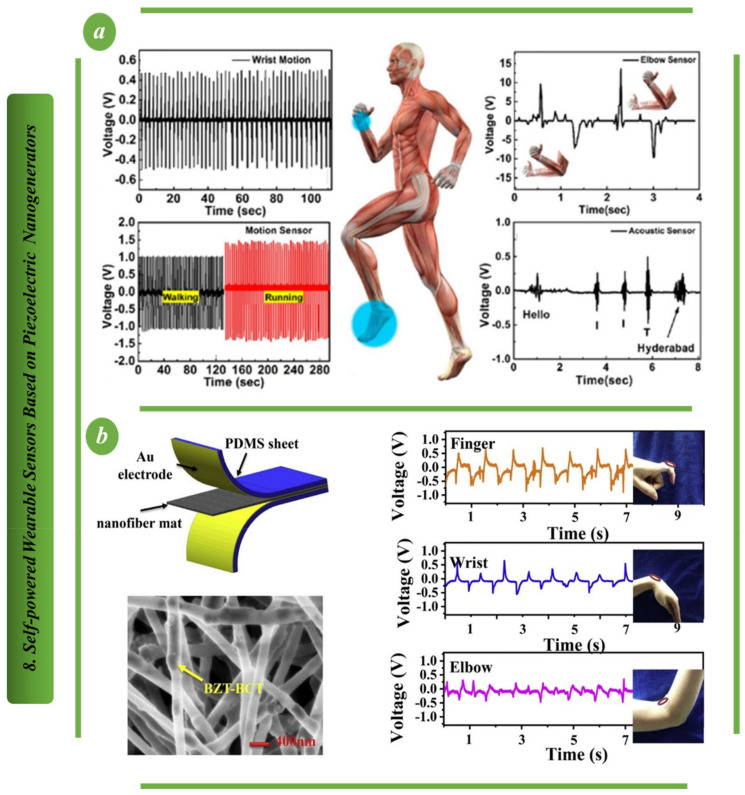
Self-powered wearable sensors based on PENG: (**a**) Illustrating the output performance of the highly flexible and fully autonomous wearable sensor based on piezoelectric nanogenerators [95]. (**b**) The principal design and output performance of wearable sensors on the finger joint, wrist joint and elbow joint based on piezoelectric nanogenerators [96].

A high-performance, flexible, biocompatible, lead-free piezoelectric nanogenerator based on bismuth tungstate (Bi2WO6) is shown in Figure 8a. It is simple and inexpensive to create biconcave-shaped Bi2WO6 nanoparticles through hydrothermal means. The robust nanogenerator’s adaptability is shown by recording acoustic signals, harvesting energy from human body motion, using it as a quick-response smart sensor door and harvesting energy from rainwater. The platform created here demonstrates its suitability for a wide range of wearable, self-powered and biocompatible electronic applications [60]. An environmentally friendly and flexible piezoelectric nanogenerator based on electrospun nanofiber is proposed in Figure 8b. The generator can be used as a self-powered sensor to track the movement of various body parts and measure tensile and compressive deformation. This newly developed nanogenerator has excellent potential for wearable and implantable devices due to its benefits of flexibility and environmental friendliness [95,96].

**Figure 9:**
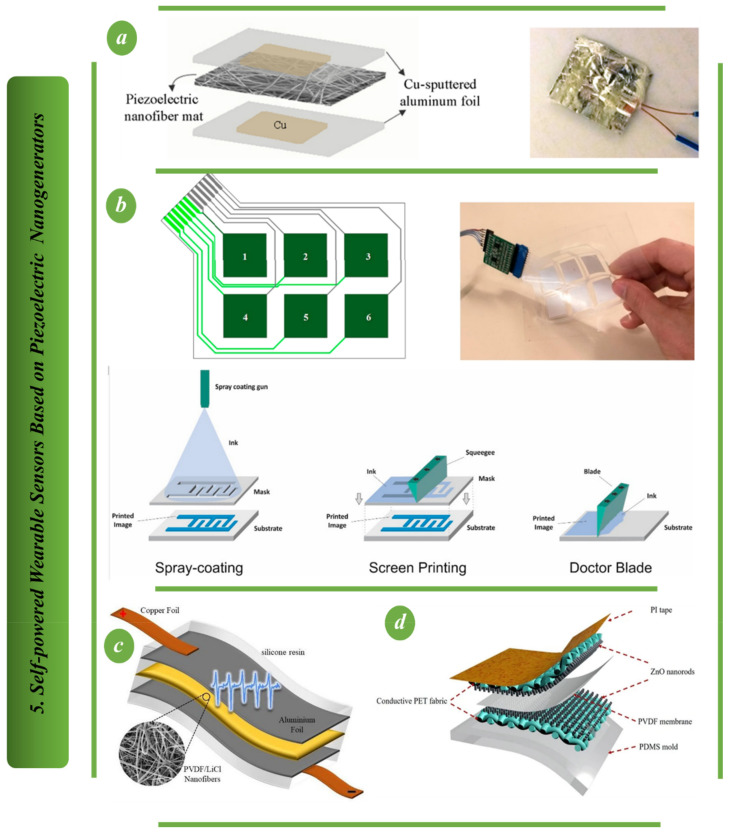
Self-powered wearable sensors based on PENG: (**a**) A unique piezoelectric sensor that may be worn and is based on a polymer (PVDF) [97]. (**b**) The mechanism of piezoelectric printed touchscreens [98]. (**c**) The polyvinylidene fluoride (PVDF)/LiCl nanogenerator device’s structural composition [99]. (**d**) The highly effective piezoelectric pressure sensor for textiles (T-PEPS) [100].

A novel wearable piezoelectric sensor for measuring arterial pulse is shown in Figure 9a and is based on a poly (vinylidene fluoride) (PVDF) nanofibrous membrane that contains microporous MOFs made of zirconium. It is demonstrated that adding 5 wt% MOF significantly increases the polymer fiber mat’s piezoelectric constant by 3.4 times without significantly reducing its flexibility. The findings of this work open up new avenues for the development of flexible piezoelectric nanofibrous sensors based on MOFs for wearable healthcare monitoring systems and ecologically sustainable energy generation [97].

The structure of a printed piezoelectric touchscreen is shown in Figure 9b. Electric films of poly (vinylidene fluoride-co-trifluoro ethylene), PVDF-TrFE, were created for touch screen application using several additive manufacturing techniques, such as doctor-blading, spray printing and screen-printing [98]. Figure 9c depicts a nanofibrous power generator in which aluminum foil was employed as the collector plate and electrospinning was performed on this substrate. Due to their high conductivity, two copper strips were adhered to both sides of the aluminum foils using silver paste, and wire was soldered onto them to link to the data acquisition board. The complete nanogenerator device was encased in silicone resin to increase its mechanical durability and shield it from dust and moisture [99]. Figure 9d depicts the creation of a ZnO nanorod-based T-PEPS. The T-PEPS has three layers: a PVDF membrane, conductive PET textiles with ZnO nanorods and a PVDF membrane. Thus, the fabricated T-PEPS features a high output voltage of 11.47 V, a low detection limit of up to 8.71 Pa and remarkable mechanical stability. The T-PEPS is employed for the detection of motions [100], including the bending and straightening of human digits and the flexing and extension of human wrists.

**Figure 10:**
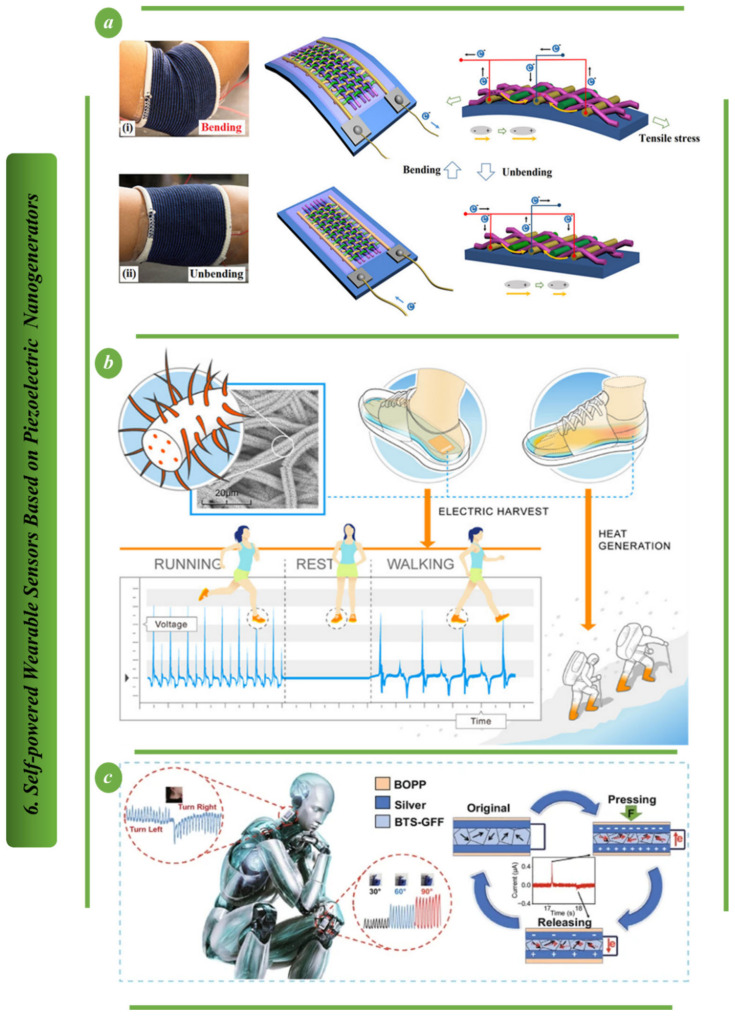
Self-powered wearable sensors based on PENG: (**a**) Illustration showing how FNG may extract usable energy from the motion of human bodies [101]. (**b**) Diagrammatic representation of the manufacturing process for the ZnO/PAN-based nano fabric that is used in the PENG [102]. (**c**) Super-flexible, lead-free piezoelectric nanogenerator for use in extremely sensitive self-powered sensors [103].

As shown in Figure 10a, a two-dimensional fabric nanogenerator based on a flexible hybrid piezoelectric fiber has the potential to be easily integrated into clothing and convert the mechanical energy of human body motion into electrical energy. Aligned BaTiO_3_ nanowires and PVC polymer made up the hybrid piezoelectric fiber. Aligned BaTiO_3_ nanowires enhanced the fiber’s piezoelectric characteristics, while the pliability of the PVC polymer made the fiber suitable for weaving. The nanogenerator with the interdigitated electrodes was created by weaving copper wires and cotton threads together. Fabric nanogenerators can produce 1.9 V output voltage and 24 nA output current when attached to a human-bent elbow pad [101]. Integration of zinc oxide nanorods (ZnO NRs) into polyacrylonitrile (PAN) nanofiber, as shown in Figure 10b, resulted in a ZnO/PAN nanofabric with approximately 2.7-times more pressure sensitivity and vibrational energy harvesting ability than that of pure PAN nanofiber. In particular, the ZnO/PAN Nano fabric produced nearly twice as much power as the ZnO and polyvinylidene fluoride fabric. The results showed that the planar zigzag conformation of PAN nanofiber microstructures was greatly enhanced by the addition of ZnO NRs [102]. Superflexible BTS-GFF/PVDF composite films and highly sensitive sensors are shown being made in Figure 10c. Medical rehabilitation, human motion tracking and intelligent robotics are just a few of the fields that stand to benefit from this structure’s novel approach to fabricating superflexible, very sensitive and wearable self-powered piezoelectric sensors [103].

## 5. Self-Powered Wearable Sensors Based on Hybrid Piezoelectric and Triboelectric Nanogenerators

**Figure 11:**
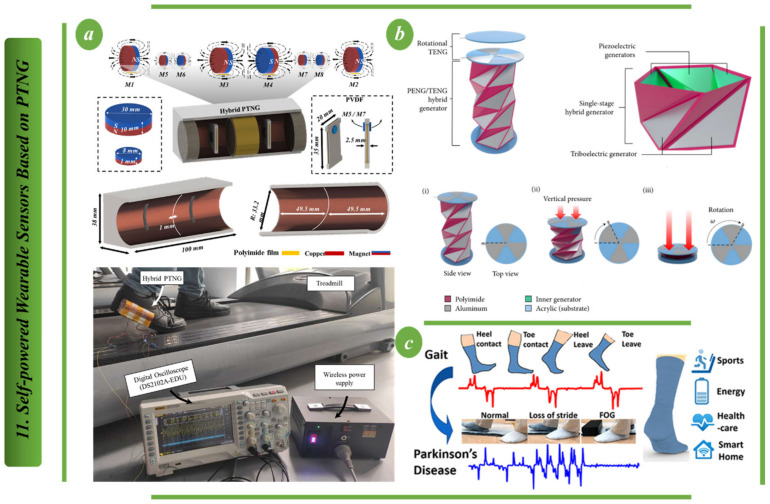
Self-powered wearable sensors based on PTNG: (**a**) Structure of the design for the hybrid PTNG that is used for walking sensing [104]. (**b**) TC piezoelectric/triboelectric hybrid generator diagram (TCO-HG) [105]. (**c**) Creating a self-powered cotton sock with piezoelectric and triboelectric nanogenerators [106].

In Figure 11, an example of a nanogenerator that combines piezoelectric and triboelectric technologies is presented. The PTNG’s fundamental copper-mode design principle is depicted in Figure 11a. The copper PTNG is made up of the copper framework, magnets on both sides and PVDF strips. The parts were 3D printed using PLA. At each end of the frames, a pair of magnets with the same magnetic polarity are fixed (M1 and M2). The length of the PVDF strip in the PTNG is also represented. Magnets of sizes M5, M6, M7 and M8 are used to hold the PVDF strips in place. PVDF strips are installed into the copper frame using the preexisting screw holes. Hybrid piezoelectric and triboelectric nanogenerators (PTNG) are shown being developed in Figure 11a for use in energy generation and monitoring. Polyimide film and copper/aluminum layers make up the triboelectric component and polyvinylidene fluoride strips in the PTNG, and magnetic force is used to apply the opposing force in the sliding mode. The maximum open-circuit voltage is found in the copper-containing triboelectric component of PTNG mode 2 (capsule with electrode layer). To better understand how people behave when exercising on a treadmill, this concept proposes a self-sufficient walking sensor system driven by PTNG. For this experiment, the design has been tested with the treadmill at different speeds [104]. Figure 11b shows an origami-based triangulated cylinder piezoelectric/triboelectric hybrid generator (TCO-HG) that can efficiently harvest energy from mechanical motion. The proposed construction has a PENG on the inner hinge, a TENG on the top substrate that rotates, and a TENG on the surface of the triangular cylinder that separates contacts vertically to harvest mechanical energy from each motion. Each generator may provide a different electrical output from the same input [105]. By combining PEDOT, a PSS-coated fabric triboelectric nanogenerator (TENG) with lead zirconate titanate (PZT) piezoelectric chips, the sock in Figure 11c is able to harvest energy and sense a variety of physiological data, such as gait, contact force, perspiration level, etc. The outputs of TENG and PZT sensors are efficiently fused together during exercise, using the concept of sensor fusion to quickly identify sweat levels [106].

Table 1 shows a summary of various piezoelectric and triboelectric nanogenerator techniques for biomedical sensors. In this table, we provided the different applications with details that we used in this review paper. 

## 6. Challenges, Perspective and Insight for Self-Powered Wearable Sensors

**Figure 12:**
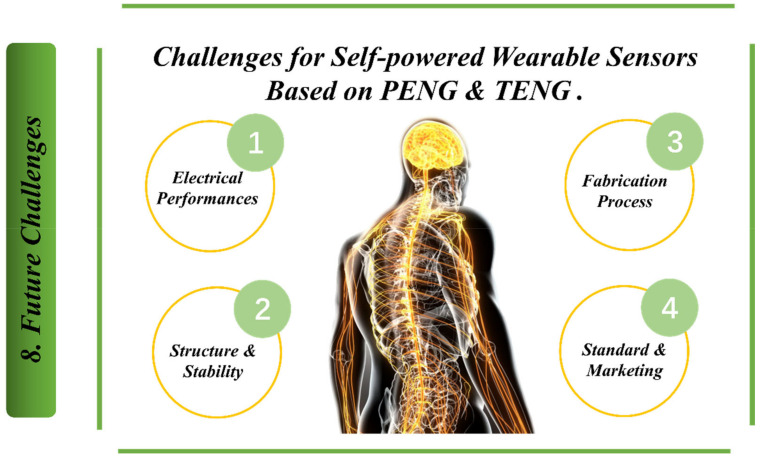
Challenges for self-powered wearable sensors based on piezoelectric nanogenerators (PENG) & triboelectric nanogenerators (TENG).

Figure 12 shows challenges for self-powered wearable sensors based on piezoelectric nanogenerators (PENG) and triboelectric nanogenerators (TENG), including:

- Electrical performance (sensing responsiveness and accuracy are the primary requirements for the sensor, with mechanical stimuli varying in intensity. Surface functionalization on the material surface and physical surface modification are favoured for superior detecting capabilities at a low detection limit, high sensitivity and rapid reaction.
- Structure and stability (to protect the functionality of active substances, harvester structures must be completely wrapped and shielded from exposure to air and sunlight).
- Fabrication process (the majority of TENGs are manually built in the laboratory in order to promote a prototype and test the viability of applications).
- In order to build a high-performance hybrid PENG-TENG, complicated composites with fillers in varying circumstances are chosen, and the charge boosting circuit is sometimes used to improve the charge density result, standard and marketing (exporting nanogenerator products necessitates client criteria and satisfaction aspects). The evaluation of standards for PENG and TENG development, such as electrical outputs, conversion, stability, and other performance parameters is unfortunately difficult to accomplish due to varying experimental and testing settings among research.
- Additionally, one of the main challenges for self-powered wearable sensors based on piezoelectric nanogenerators (PENG) & triboelectric nanogenerators (TENG) is power output. In fact, researchers are trying to increase the current for this concept through magnetic induction. This method will play a key role in the future of smart sensors based on the triboelectric and piezoelectric technology. Another challenge that researchers are attempting to overcome is the development of a new form of effective self-powered mechanical sensor, based on hybrid nanogenerators based on the flexoelectric effect for wearable sensors. The flexoelectric effect can be regarded as a novel approach to the development of high-performance self-powered mechanical sensors. The flexoelectric effect is caused by interactions between electrical polarization and a strain–stress gradient, and it fundamentally entails the development of an electric polarization response or a mechanical reaction under a mechanical strain–stress gradient. Furthermore, unlike the piezoelectric effect, which is exhibited in particular materials with structurally asymmetric crystal structures, the flexoelectric effect can occur even in dielectric materials with symmetric crystal structures due to induction by mechanical gradients. As a result, unlike the piezoelectric effect, the flexoelectric effect’s electric polarization can be improved by maximizing the strain–stress gradient through the control of various nano or microscale materials and devices, which can be applied to all-dielectric materials to effectively generate electrical signals from mechanical stimuli.

## 7. Conclusions

In summary, a self-powered wearable sensor based on piezoelectric and triboelectric nanogenerators is demonstrated for health care systems. Smart sensors are advantageous because they can be produced quickly and cheaply, worn conveniently, have a long lifespan, respond quickly, are resistant to water and other elements and may be used at any time and in any location. The development of PENGs and TENGs has been a significant breakthrough in the study of self-sufficient sensor technology for wearable devices. In the last few years, tremendous progress has been made with regard to self-powered wearable sensors in the application sectors of medicine and healthcare. Self-powered flexible sensors have the potential to successfully overcome the drawbacks of a battery that has a limited lifetime, in comparison with conventional self-powered wearable sensors which are powered by an external battery. In addition, TENGs and PENGs have the potential to eliminate the uncertainty associated with energy conversion in particular places. In this review, smart wearable sensors for a health monitoring system based on triboelectric nanogenerators (TENG), piezoelectric nanogenerators (PENG) and hybrid piezoelectric and triboelectric nanogenerators were discussed. We first introduced the PENG and TENG principles, and then we summarized and discussed the most recent developments in self-powered medical information sensors with various functionalities, structural designs and electric performances.

## Figures and Tables

**Table 1:** Summary of various piezoelectric and triboelectric nanogenerators techniques for biomedical sensors.

Structure	Year	Authors	Applications	Max-Open-Circuit Voltage (V)	Max-Short- Circuit Current	Surface Power Density	Power Density and Power	Advantages/Disadvantages	Dimensions
Triboelectric nanogenerators	2021	Qin et al. [83]	Sensor for real-time gesture interaction	~2 V	-	-	-	Simple structure, high power density and low-frequency/lower durability, limited short circuit output current, structural changes	20$\times 12{\;\mathrm{mm}}^{2}$
	2021	Zhang et al. [84]	Wearable sensor	~50 V	-	-	-		210 mm ×297 mm
	2017	Li et al. [85]	Wearable TENG for high performance biomedical energy harvesting	540 V	110 µA	-	-		$16{\;\mathrm{cm}}^{2}$
	2015	Kim [86]	Wearable TENG under harsh environments	40 V	210 µA	-	4 mW		0.5 cm × 0.5 cm
	2020	Bai et al. [87]	Efficient energy harvesting and motion sensing	~158 V	15 µA	2.5 mW/m^2^	-		-
	2022	Ying et al. [88]	Wearable sensor and biomedical energy harvesting	~200 V	-	5.7 mW/m^2^	-		3$\times 3{\;\mathrm{cm}}^{2}$
	2022	Yi et al. [89]	Wearable sensing system for real-time vital signs monitoring	~450 V	~25 µA	~816.6 mW m^−2^	-		4 cm × 4 cm
	2019	Lin et al. [90]	Biomechanical sensor	~550 V	~80 µA	-	5.47 mW		-
	2021	Li et al. [91]	Wearable electronics sensor	~140 V	~2 µA	-	-		45 cm × 5 cm × 2 mm
	2020	Liu et al. [92]	Stretchable motion sensor	38 V	-	-	-		-
Piezoelectric nanogenerators	2019	Zhang et al. [93]	Sensing	~25 mV	-	-	-	Low internal resistance/complex structure	-
	2021	Petritz et al. [94]	Imperceptible energy harvesting device and biomedical sensor	~3.5 V	-	3 mW·cm^−3^	-		3 cm × 2.5 cm
	2022	Veeralingam et al. [95]	Wearable electronic sensors and energy harvesting through rainwater	50 V	0.6 μA/cm^2^	-	-		-
	2020	Liu et al. [96]	Human motion sensing	13.01 V	-	-	1.44 µW		-
	2020	Moghadam et al. [97]	Arterial pulse monitoring	~568 mV	-	-	-		3$\times 3{\;\mathrm{cm}}^{2}$
	2019	Gonçalves et al. [98]	Touch screen technologies	5 V	-	-	-		20$\times 30{\;\mathrm{cm}}^{2}$
	2020	Mokhtari et al. [99]	Self-powered wearable technologies	3 V	0.5 µA	0.3 µW/m^2^	-		3 cm × 1.5 cm
	2021	Tan et al. [100]	for wearableapplication	11.47 V	-	-	-		-
	2015	Zhang et al. [101]	For wearable sensors	1.9 V	24 nA	-	10.02 nW		-
	2020	Sun et al. [102]	Energy harvesting for motion sensing	-	-	10.8 mW/m^2^	-		20 cm × 9 cm
	2021	Yu et al. [103]	Self-powered sensor for human motion monitoring	~25 V	~0.5 µA	-	-		2$\times 3{\;\mathrm{cm}}^{2}$
Hybrid Piezoelectric and Triboelectric Nanogenerators	2022	Matin Nazar et al. [104]	Walking sensing	21.9 V	-	-	70 µW	High output performance/limitation of applications	100 mm × 38 mm× 22 mm
	2021	Chung et al. [105]	Sensing applications	120 V	90 µA	-	-		20 cm × 25 cm
	2019	Zhu et al. [106]	Healthcare and sports monitoring	200 V	~6 µA	128 mW/m^2^	1.71 mW		5 mm × 5 mm

## Data Availability

The data presented in this study are available on request from the corresponding author.
